# Supplementary material for: CT Perfusion as a Predictor of the Final Infarct Volume in Patients with Tandem Occlusion
Source: J Pers Med. 2023 Feb 16;13(2):342. doi: 10.3390/jpm13020342 (PMC9964425; doi:10.3390/jpm13020342)
Supplement: Supplementary file 1 [file jpm-13-00342-s001.zip › jpm-2142759-supplementary.pdf]

CT perfusion as predictor of the final infarct volume in patients with tandem occlusion

Giordano Lacidogna [1,*], Francesca Pitocchi [2], Alfredo Paolo Mascolo [1], Federico Marrama [1], Federica D'Agostino [1], Alessandro Rocco [1], Francesco Mori [1], Ilaria Maestrini [1], Federico Sabuzi [3], Armando Cavallo [2], Daniele Morosetti [3], Francesco Garaci [2], Francesca Di Giuliano [2], Roberto Floris [2], Fabrizio Sallustio [1], Marina Diomedi [1] and Valerio Da Ros [3]

Affiliation 1; Stroke Center, Department of Systems Medicine, University Hospital of Rome "Tor Vergata", Viale Oxford 81, 00133, Rome, Italy

Affiliation 2; Diagnostic Imaging Unit, Department of Biomedicine and Prevention, University of Rome Tor Vergata, Viale Oxford 81, Rome, 00133, Italy.

Affiliation 3; Department of Biomedicine and Prevention, University Hospital of Rome "Tor Vergata", Viale Oxford 81, Rome, Italy

* Correspondence: Giordano Lacidogna g.lacidogna@libero.it; Tel.+390620903423

Supplemental material

Results

- 83 patients (26%) were treated for concomitant extracranial ICA and MCA occlusion (*Tandem group-TG*) and in 49 cases (59%) successfull recanalization (mTICI 2b-3) was reached. 29 subjects (34,9%) underwent a CTP perfusion study implemented with RAPID software. 5 patients (17,2%) had a wake up stroke and 1 patient (3.44%) had an unknown onset time stroke. 6 subjects (24.1%) showed altered perfusional AIF and VOF curves and were excluded from the analysis; 1 patient in the tandem group experienced an intracranial re-occlusion and was excluded from the analysis as per inclusion criteria (Figure S1 Suppl. Mat.). No embolism in a new vascular territory, vasospasm, and femoral artery access injuries occured in the sample. No occlusion in anterior cerebral artery were reported.

  Among 167 patients (52,3%) who experienced an AIS exclusively due to a MCA occlusion 146 reached successful recanalization (45,8%) (*Control Group* - CG). Among them, 42 controls were selected according to the execution of a CT perfusion study with RAPID software before treatment. Among these, 5 patients (11,9%) with altered perfusional AIF and VOF curves were excluded. 10 patients (23.8%) had a wake up stroke, 3 patients (7.1%) had an unknown onset time stroke (Figure S1).

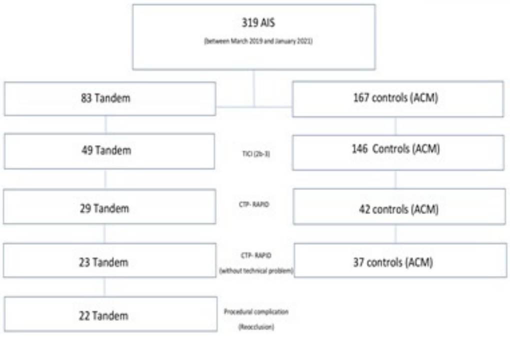

Figure S1. recruitment flow chart

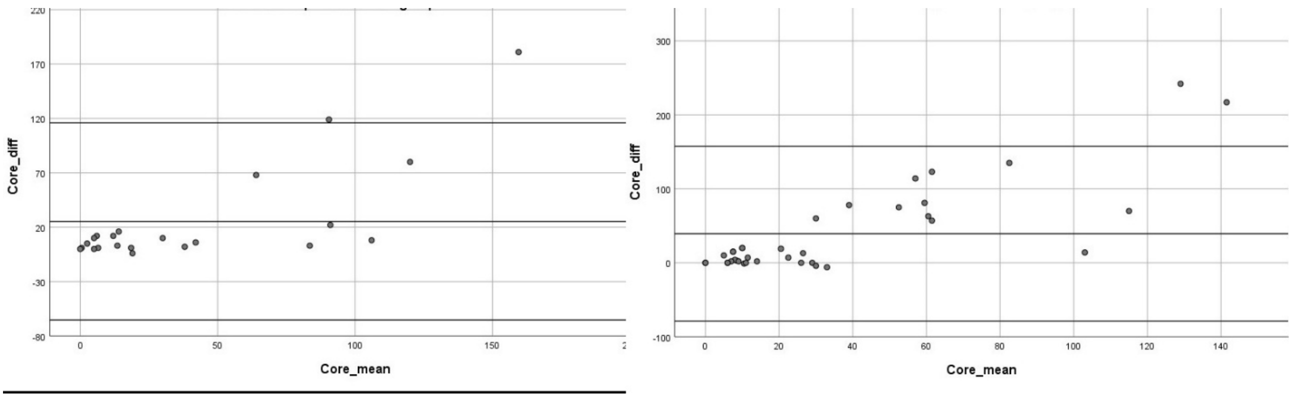

Figure S2 Distribution of mRS outcome.

- To control for potential effects of minor hemorrages on FIV we performed a one-way ANOVA, controlled with Brown-Forsythe, due to the lack of homogeneity of variance among groups. A significant difference was found between groups (F= 15,73, between groups df= 4, within groups df=54, p<0,0001). Tukey's HSD Test for multiple comparisons found that the mean value of FIV was significantly different between PH2 group and all other groups (p<0,0001, 95% CI=53,14-115,88 with NO-ICH, p< 0,001, 95%CI= 23,77- 122,14 with HI1 group, p< 0,0001 95%CI = 42,37-140,74 with HI2 group, p< 0,001 95% CI= ,91- 84,90 with PH1group) and between PH1 group and NO-ICH group (p= 0,022 95%CI= 4,14-79,08). Notably no significant difference was detected between PH1 group, HI1 group and H2group. The same significant differences remain when controlling for potential effect of minor hemorrages on differences between PIC and FIV. Means and standard deviation of FIV and differences between PIC and FIV were reported in Table S1.

Table S1. Means and standard deviations of FIV and differences between PIC and FIV among hemorrhage subtypes

	subgroup	N	mean	SD
FIV	No ICH	28	22,64	23,747
	HI1	4	34,2	28,839
	HI2	7	15,6	6,189
	PH1	7	64,25	44,88
	PH2	13	107,15	46,925
FIV-PIC difference	No ICH	28	9,32	20,692
	HI1	4	9,6	10,502
	HI2	7	7,6	6,025
	PH1	7	39	37,603
	PH2	13	76,23	47,078

ICH: intracerebral hemorrhages; HI: hemorrhagic infarction;PH: parenchymal hematoma

- A Kruskal-Wallis Test for non parametric indipendent variable was conducted in the whole sample to examine the differences between mRS outcomes according to the types of hemorragic trasformation. A significant effect of hemorrage type (Chi square = 23.72, p = .000, df = 4) was found on mRS outcome. A post hoc analysis with Bonferroni correction showed a significant difference between NO ICH and PH1 group (Chi square = -26.72, p = .021), and between PH2 and NO-ICH group

(Chi square -26,26 p=.000, for HI1, Chi square -18,64 p=.041). No significant differences were found among PH2 and PH1 and PH2 and H2 fo the main outcome.

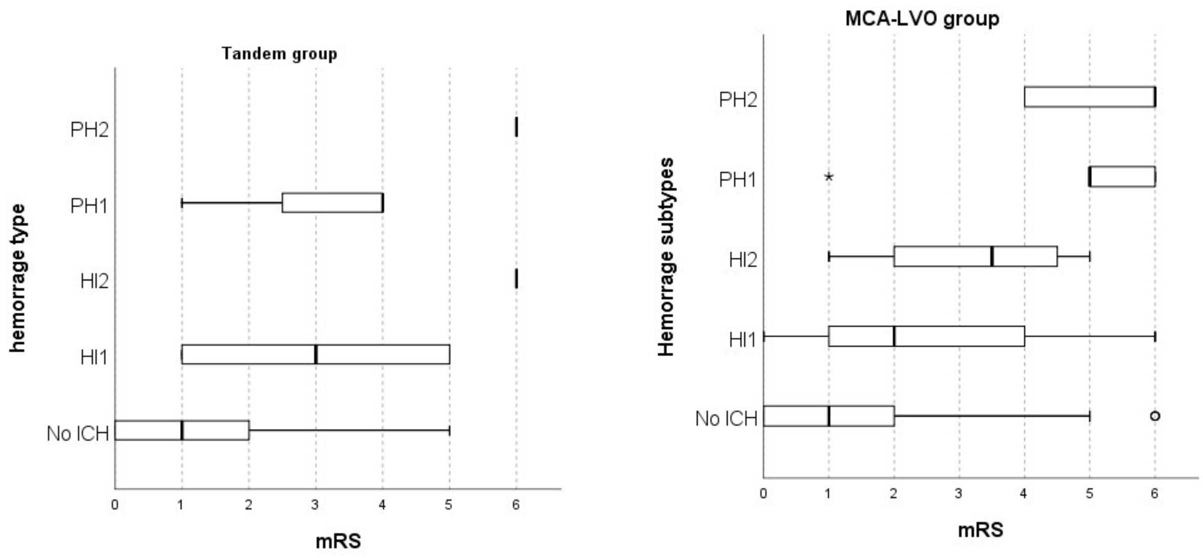

Figure S3. Oucome distributions between different subtypes of postprocedural hemorrhagic trasformation

- *Secondary analysis performed by excluding patient who suffered a hemorrhagic trasformation with mass effect (PH2) and partially recanalized patients (TICI 2b or 2c)*

Table S2. Comparisons between groups for baseline features.

	Tandem (18)	MCA-LVO (28)	*p*
Gender, n (%)			
Male	10 (52,6)	12 (43,3)	.569
Female	8 (47,4)	16 (56,7)	.569
Age, median (IQR)	67 (15)	73,5 (17)	.192
Pre stroke mrs, median (IQR)	0 (0)	0 (0)	> 0,05
Arterial Hypertension, n (%)<br>Missing 1	16 (84,2)	21 (70)	.323
Atrial Fibrillation, n (%)<br>Missing 1	7 (368)	12 (40)	.852
Hypercholesterolemia (%)<br>Missing 1	4 (21,1)	7 (23,3)	.825
Diabetes, n (%)<br>Missing 1	2 (10,5)	3 (10)	.953
Smoking, n (%)<br>Missing 1	3 (15,8)	5 (16,7)	.935
Alcohol (%)<br>Missing 1	2 (10,5%)	0	.145
Etiology - TOAST (%):**			.057
Undetermined	3 (15,8)	10 (33,3)	
Large artery	9 (52,6)	5 (16,7)	
Cardioembolic	4 (21,1)	10 (40)	
Other	2 (10,5)	3 (10)	
ASPECTS, median (IQR)	9 (2)	10 (2)	.097
Good collateral Score, n (%)	13 (68,4)	22 (73,3)	.754

NIHSS, median (IQR)	19 (7)	15,5 (10)	.677
Clot Burden score, median (IQR)	4 (2)	6 (2)	**<.001**
Occlusion site, n (%)			.524
M1 prox	14 (78,9)	21 (70)	
M1 dist	0	3 (10)	
M2	4 (21,1)	6 (20)	
Occlusion side,n (%)<br>left	 9 (50)	 14 (46,7)	.596
right	9 (50)	16 (53,3)	
Systolic Blood pressure<br>Missing 2C+2T	144 (26,97)	129,54 (18,10)	
Diastolic blood pressure<br>Missing 2C+2T	91 (20,49)	74,11 (10,54)	
Glycemia<br>Missing 2C+2T	117, 11 (40,34)	117,11(27,62)	

Table S3. Procedural times and strategies.

	Tandem (n=18)	Controls (n=28)	*P*
Onset to groin (SD)<br>Missing**: 4T+12C	262,36 (198,04)	253,22 (122, 17)	.129
Onset to CTP (SD)<br>Missing: 4T+12C	207,00 (209,08)	189,00 (113,20)	.123
CTP to recanalization (SD)<br>Missing:	129,74 (42,46)	102,7 (34,07)	**.035**
Onset to reperfusion, mean (SD)<br>Missing: 4T+12C	330,5 (208,55)	298,22 (121,11)	.343
Procedural time, mean (SD)<br>Missing:	66,32 (49,60)	36,93 (23,62)	**.016**
Rtpa, n (%)	6 (31,6)	6 (20)	.468
General anesthesia	0	2 (6,7)	.515
Passages:<br>Median (IQR)	2 (2)	1 (1)	**0.03**
Technique:<br>Thromboaspiration (%)	 11 (62,2)	 28 (93,3)	.019*
Stent retriever (%)	0	0	
Solumbra (%)	7 (36,8)*	2 (6,7)	
